# Low-dose cyclosporine in treatment of membranous nephropathy with nephrotic syndrome: effectiveness and renal safety

**DOI:** 10.1080/0886022X.2017.1373130

**Published:** 2017-09-08

**Authors:** Xiaojuan Yu, Lin Ruan, Zhen Qu, Zhao Cui, Yimiao Zhang, Xin Wang, Liqiang Meng, Xiaojing Liu, Fang Wang, Ying Zhang, Gang Liu, Li Yang

**Affiliations:** a Renal Division, Department of Medicine, Peking University First Hospital, Beijing, P.R. China;; b Institute of Nephrology, Peking University, Beijing, P.R. China;; c Key laboratory of Renal Disease, Ministry of Health of China, Beijing, P.R. China;; d Key Laboratory of CKD Prevention and Treatment, Ministry of Education of China, Beijing, P.R. China;; e Renal Division, Department of Medicine, First Municipal Hospital, Shijiazhuang, Hebei province, P.R. China

**Keywords:** Cyclosporine, membranous nephropathy, nephrotic syndrome, nephrotoxicity, renal injury

## Abstract

**Background:** To observe effectiveness and renal safety of long-term low-dose cyclosporine in idiopathic membranous nephropathy (IMN).

**Methods:** Sixty-eight patients were enrolled in this prospective cohort study. Renal endpoint was defined as a decrease in eGFR ≥50% from baseline and a development of eGFR ≤60 ml/min/1.73m^2^.

**Results:** A cyclosporine dose of 2.0 ± 0.5 mg/kg/d and a prednisone of 0.3 ± 0.2 mg/kg/d were prescribed. The duration of cyclosporine treatment was 27 (3–80) months. The overall remission rate was 91% with a relapse rate of 42%. Fourteen patients had cyclosporine-related acute renal injury (CsA-ARI) within the first three months, and 16 patients had cyclosporine related chronic renal injury (CsA-CRI) within the first year. At the end of follow-up (50 ± 18 months), 16 patients (24%) reached renal endpoint. Presence of intimal fibrosis of small artery and higher time-averaged proteinuria were identified as independent risk factors for renal endpoint. RAS inhibition treatment decreased the risk of poor renal outcome. Patients in CsA-ARI group had the highest proteinuria at the third month, the highest time-average proteinuria and the highest proportion of cases reaching renal endpoint. Patients with CsA-CRI were of the oldest age and with the lowest baseline eGFR.

**Conclusions:** Low-dose cyclosporine is effective in treating IMN. CsA-ARI and no response in proteinuria during the first three months of cyclosporine treatment had the lowest benefit/risk ratio, and these patients should be switched to non-calcineurin-inhibitor based regimen. Patients of older age, with lower baseline eGFR, or having intimal sclerosis of small artery, are more likely to develop progressive renal dysfunction.

## Introduction

Idiopathic membranous nephropathy (IMN) is the most common cause of nephrotic syndrome in adults and accounts for 15–36% of the renal biopsies [1–3]. Treatment of IMN with immunosuppressive agents is mainly based on the balance among risks of disease progression and complications, effectiveness of immunosuppressive agents, and the associated side effects. Cyclosporine has been proven to be as effective as alkylating agents in treating IMN [4–6], whereas stopping cyclosporine treatment is correlated with high relapse rates, consequently leading to long-term use. One of the major concerns about cyclosporine is renal injury, which, according to previous studies, is dose and cumulative usage time dependent [7,8]. In an attempt to decrease side effects, lower dosage of cyclosporine (1.5–3 mg/kg/d) had been tried in a few small sample size studies and found effective in treating IMN with remission rates of 57.1–100% [9–14]. Yet, reports documenting the nephrotoxicity associated with prolonged low-dose cyclosporine are conflicting [9,10], and long-term follow-up studies are still limited.

In this prospective cohort study, 68 IMN patients were prescribed low initial dose of cyclosporine (1.0–1.5 mg/kg/d) and prednisone (0.15–0.50 mg/kg/d) and followed-up for 50 ± 18 months. The remission rate, relapse rate, short-term and long-term renal safety, as well as risk factors for renal injury were investigated.

## Methods

### Patients

**Figure 1.**
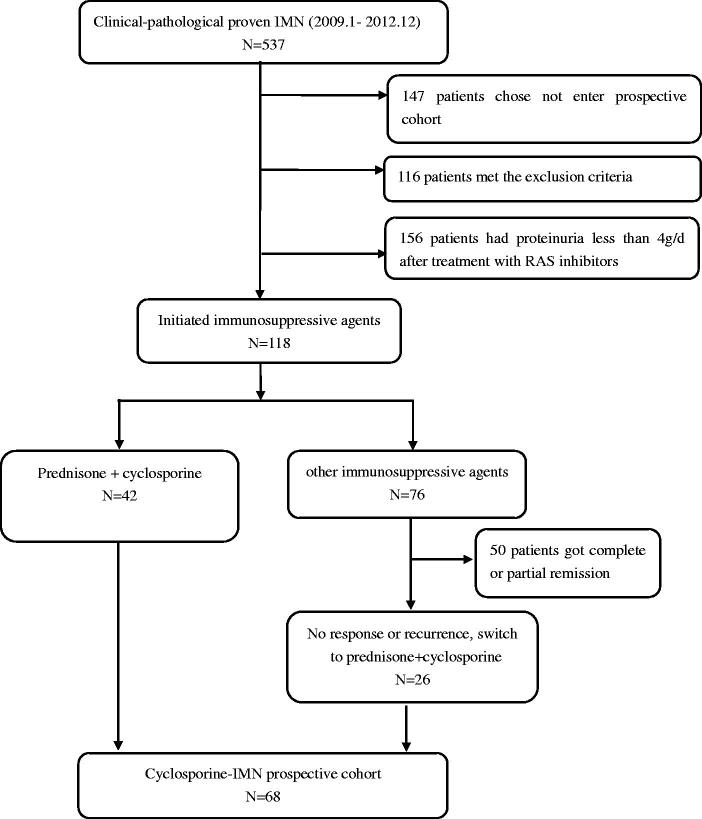
Patients recruitment flowsheet.

Adult patients with newly biopsy-proven IMN from 2009 to 2012 in Peking University First Hospital were prospectively screened. Patients meeting the following criteria were included: (1) renal biopsy confirmed MN and exclusion of secondary causes through detailed clinical history and laboratory tests. (2) Treatment failure after renin-angiotensin-aldosterone (RAS) inhibitors or corticosteroid and other immunosuppressive agents. The details for recruitment are shown in Figure 1.

Patients with any of the following conditions were excluded: (1) Superimposed with other glomerular diseases or tubulointerstitial diseases. (2) Serum creatinine (SCr) ≥ 133 μmol/L or estimated glomerular filtration rate (eGFR) ≤ 60 mL/min/1.73m^2^, or ≥50% of cortex showing chronic tubulointerstitial changes in renal biopsy. (3) Steroid or cyclosporine treatment contraindications such as uncontrolled hypertension or diabetes mellitus, etc. (4) Accompanied with systemic diseases such as severe cardiovascular disease, hepatic impairment, etc. (5) Pregnancy or women unwilling to take effective birth control measures. (6) Unwilling to use immunosuppressive agents. (7) Unable to enter the prospective follow-up program or unwilling to join the study.

Informed consent was obtained from each patient. The research was in compliance with the declaration of Helsinki and approved by the ethic committee of Peking University First Hospital.

### Cyclosporine treatment protocol

The initial cyclosporine dose was 1.0–1.5 mg/kg/d in two divided doses combined with prednisone at 0.15–0.50 mg/kg/d [11]. The wide range of initial prednisone dose was due to some patients were switched from non-calcineurin inhibitors-based first treatment regimen. Cyclosporine doses were adjusted monthly according to efficacy or targeting a whole blood 12 h trough level of 75–150 ng/ml [11,15]. In patients who achieved complete/partial remission, the dose was gradually tapered to the lowest dose maintaining remission. Cyclosporine treatment was accomplished when patients maintained complete/partial remission for one year [16].

Renal injury was defined if SCr increased by ≥30% or eGFR decreased by ≥30% from baseline at any time during the first year of cyclosporine initiation, and cyclosporine dose was reduced by 25% if patients had achieved complete/partial remission, or SCr had increased to ≥133 μmol/L. Cyclosporine was discontinued if patients had any of the followings: (1) SCr maintained ≥30% increase from baseline and ≥133 μmol/L after decreasing cyclosporine dose by 50%; (2) proteinuria remained at ≥50% of baseline and ≥3.5 g/d after 6 months of cyclosporine; (3) developed life-threatening infections or intolerable side effects; (4) reluctant to continue cyclosporine treatment due to side effects or any other reasons.

RAS inhibitors were administered to all the tolerated patients.

### Clinical and laboratory assessment during follow-up

Data at the time of cyclosporine commencement (baseline data) were collected. eGFR was calculated using the Epidemiology Collaboration equation [17,18]. Patients were followed-up in outpatient clinic every month during the first 6 months, and every 3 months or more often if needed after 6 months. At each visit, clinical data and serum cyclosporine levels were collected. Any side effects that might be related to treatment were documented.

Complete remission (CR) was defined as proteinuria ≤0.3 g/d, confirmed by two values at least one week apart, accompanied by normal serum albumin. Partial remission (PR) was defined if patients’ proteinuria maintained at 0.3–3.5 g/d and/or ≥50% reductions from peak values which was confirmed by two values at least one week apart, accompanied by an improvement or normalization of serum albumin. Relapse was defined as an increase of proteinuria to ≥1 g/d in CR patients, or a doubling of proteinuria to levels ≥1 g/d in PR patients.

Time-average proteinuria (TAP) was calculated by the area under the curve of proteinuria during whole follow-up course divided by months [19]. The renal endpoint of the current study was defined if eGFR decreased by ≥50% from baseline and <60 mL/min/1.73m^2^.

### Renal histopathology assessment

Renal biopsy was examined by routine direct immunofluorescence, light microscopy and electron microscopy. Two pathologists evaluated biopsies separately. Differences in scoring between the two pathologists were resolved by re-reviewing the biopsies to reach a consensus.

IMN was staged as I to IV according to the classification of Ehrenreich and Churg [20]. Semi-quantitative scores were made for acute tubular injury, tubular atrophy, interstitial infiltrates, and interstitial fibrosis, according to the percentage of affected tubulointerstitial compartment: ‘0’ for 0–25%, ‘1’ for 25-50% and ‘2’ for >50%. Acute tubulointerstitial index was made by adding up the scores of acute tubular injury and interstitial infiltrates; chronic tubulointerstitial index was calculated as the sum of tubular atrophy and interstitial fibrosis. Artery/arterioles lesions, including intimal sclerosis of small artery and arteriole hyalinosis, were documented as ‘0’ for absence and ‘1’ for presence.

### Statistical analysis

Statistical software SPSS 13.0 (SPSS, Chicago, IL) was used. Continuous data were expressed as mean ± s.d, or median with range. Categorical variables were presented as proportions. Differences among groups were compared using ANOVA for normally distributed and homogeneous quantitative data, Kruskal–Wallis H test for non-normally distributed, heterogeneous quantitative and semiquantitative data, and Chi-square or Fisher’s exact test for polychotomous data. Possible indicators for renal injury and renal endpoint were first analyzed by univariate Cox regression followed by multivariate Cox regression. Results were expressed as hazard ratio with 95% confidence intervals. All *p* values are two-sided, and statistical significance was considered as *p* < .05.

## Results

### Demographic and general clinical-pathological data

Sixty-eight patients, 38 male (56%) and 30 female (44%), were enrolled in the cyclosporine-IMN prospective cohort. Renal biopsy confirmed IMN stage I in 36 patients (53%), stage II in 26 patients (38%), and stage III in 6 patients (9%). The mean acute and chronic tubulointerstitial index was 1.2 ± 0.7 (median: 1; range 0–3) and 1.3 ± 0.8 (median: 1; range 0–2), respectively, with commonly seen artery/arteriole changes (57%, 39/68).

At the time of cyclosporine commencement, the mean age was 53 ± 14 years. SCr were 73.3 ± 15.6 μmol/L, and eGFR were 103.9 ± 25.2 mL/min/1.73m^2^. Forty-seven patients (69%) were at CKD-1 and the rest (31%) were at CKD-2. Serum albumin was 27.5 ± 6.0 g/L with proteinuria of 5.4 (1.9–21.3) g/d. Concurrent comorbidities included hypertension in 23 patients (34%) and type 2 diabetes mellitus in 10 patients (15%).

### Effectiveness of treatment

A maximum cyclosporine dose of 2.0 ± 0.5 mg/kg/d was prescribed to reach a whole trough cyclosporine blood level of 105.2 ± 46.8 ng/ml. The dose of prednisone was 0.3 ± 0.2 mg/kg/d. RAS inhibition treatment was maintained in 57 patients (84%). Altogether, 91% patients (62/68) achieved PR at 4.5 (1–12) months, among which 29 achieved CR at 12.5 (4–49) months, whereas 26 patients (42%, 26/62) had relapse, including 21 patients got relapse during cyclosporine maintenance at a dose of 95 ± 38 mg/d and 5 patients at 14 (1–19) months after stopping cyclosporine. Cyclosporine dosage was increased in 14 patients or restarted in three, among which 12 patients (70%, 12/17) responded again. The rest 9 patients with relapse switched to other immunosuppressive agents (including tacrolimus, cyclophosphamide, mycophenolate mofetil, leflunomide). By the end of follow up (50 ± 18 months), the overall remission rate of this prospective IMN cohort remained at 82% (56/68), and 15 patients (22%, 15/68) were still on cyclosporine. The overall time of cyclosporine treatment was 27(3–80) months.

### Renal function changing profile

**Figure 2.**
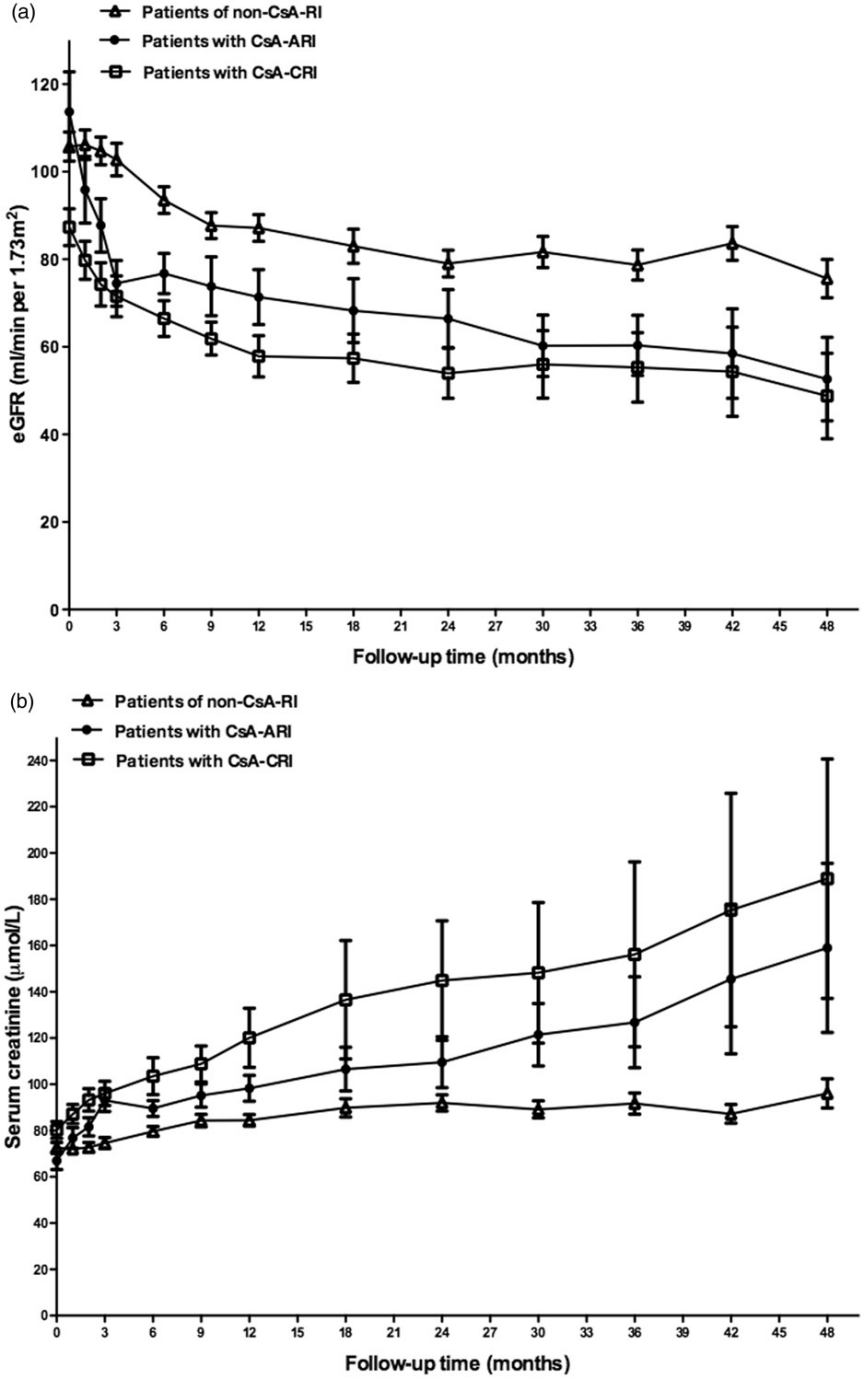
(a) The eGFR change in patients with Non-CsA-RI, CsA-ARI and CsA-CRI during the first four years of follow-up. (b) The SCr change in patients with Non-CsA-RI, CsA-ARI and CsA-CRI during the first four years of follow-up.

According to the changes in SCr and eGFR during the first year, particularly within the first three months, there were three different types of renal function changing profile (Figure 2(a,b)). Fourteen patients (20%) presenting relatively rapid renal injury (SCr increased by ≥30% and/or eGFR decreased by ≥30% of baseline levels) during the first three months were defined as ‘cyclosporine related acute renal injury’ (CsA-ARI). Sixteen patients (24%) had sub-rapid decrease in renal function with SCr started to increase during the first three months but reached the diagnostic criteria for renal injury within the left 9 months of the first year, and were defined as ‘cyclosporine related chronic renal injury’ (CsA-CRI). The rest patients (*n* = 38, 56%) had stable renal function within the first three months and had SCr increased by <30% during the first year, and were defined as ‘no cyclosporine related renal injury’ (Non-CsA-RI). Five patients discontinued cyclosporine due to SCr remained ≥133 μmol/L after dose decrease.

### Comparisons of clinical-pathological features among patients with different renal function changing profile

As shown in Table 1, patients in Non-CsA-RI group had the highest overall remission rate and the lowest TAP among the three groups. Those who developed CsA-ARI had the highest baseline eGFR, older age, more frequency of artery lesions, and higher peak serum cyclosporine levels, whereas the differences did not reach statistical significance. During the follow-up course, patients with CsA-ARI had the highest proteinuria at 3rd month (ARI 5.98 ± 4.28 vs. CRI 3.65 ± 2.31 vs. non-CsA-RI 3.84 ± 2.66, *p* = .183) and the highest TAP among the three groups. By multivariate Cox hazard analysis, proteinuria at the time of CsA-ARI was identified as an independent risk factor for CsA-ARI (HR =1.173, 95% CI 1.029–1.337, *p* = .017). Patients with CsA-CRI were of the oldest age and with the lowest baseline eGFR levels among the three groups (Table 1). By multivariate Cox hazard analysis, baseline eGFR was the independent risk factor for the development of CsA-CRI (HR =0.962, 95% CI 0.933–0.992, *p* = .012).

**Table 1.** Comparison on demographic data and general data among non-CsA-RI, CsA-ARI and CsA-CRI groups.

	Non-CsA-RI (*n* = 38)	CsA-ARI (*n* = 14)	CsA-CRI (*n* = 16)	*p* Value
Baseline data at CsA commencement				
Age (years)	51 ± 15	57 ± 12	59 ± 10	.07
Male, *n* (%)	24 (63)	7 (50)	7 (44)	.37
Mean blood pressure (mmHg)	93 ± 10	92 ± 7	96 ± 11	.60
Serum creatinine (µmol/L)	72.5 ± 15.2	67.0 ± 14.3	8.3 ± 14.5	.05
eGFR (ml/min/1.73 m^2^)	105.8 ± 21.8	113.7 ± 34.2	87.4 ± 16.80	.008
CKD stage 1:stage 2 (*n*)	30:8	11:3	6:10	.012
Serum albumin (g/L)	27.9 ± 6.1	25.8 ± 5.3	28.7 ± 5.6	.38
Proteinuria (g/d)	6.1 ± 3.9	7.10 ± 3.4	6.0 ± 2.6	.39
Hypertension, *n* (%)	11 (29)	5 (36)	7 (44)	.73
Diabetes mellitus, *n* (%)	5 (13)	3 (21)	2 (12)	.81
Renal pathology				
Stage of IMN (I:II + III) (*n*)	27:11	5:9	4:12	.003
Tubule/interstitial acute index	1.2 ± .8	1.1 ± .7	1.3 ± .7	.73
Tubule/interstitial chronic index	1.3 ± .8	1.4 ± .7	1.2 ± .8	.82
Artery/arteriole lesions, %(*n*/*n*)				
Intimal sclerosis of small artery	50 (19/38)	50(7/14)	57 (8/14)	.89
Hyalinosis of arteriole	27 (10/37)	50 (7/14)	38 (6/16)	.30
Treatment				
RAS inhibition, *n* (%)	35 (92)	11 (79)	11 (69)	.071
Initial prednisone dose (mg/kg/d)	.4 ± .2	.3 ± .2	.3 ± .1	.28
Initial CsA dose (mg/kg/d)	1.6 ± .5	1.6 ± .4	1.6 ± .3	.88
Maximum CsA dose in first 3 m (mg/kg/d)	2.0 ± .3	2.0 ± .4	2.1 ± .4	.72
Peak serum CsA level in first 3 m (ng/ml)	99.8 ± 39.9	129.5 ± 6.5	111.8 ± 54.5	.22
By the end of first year				
Serum creatinine (µmol/L)	86.0 ± 18.4	92.2 ± 18.6	106.1 ± 17.2	.003
eGFR (ml/min/1.73 m^2^)	86.0 ± 19.8	76.7 ± 21.2	62.3 ± 14.5	.001
ΔeGFR (ml/min/1.73 m^2^)	16.1 ± 16.1	42.3 ± 19.7	29.5 ± 16.6	<.001
Serum albumin (g/L)	37.2 ± 5.7	34.9 ± 7.1	36.8 ± 6.9	.519
Proteinuria (g/d)	1.92 ± 2.31	4.84 ± 4.43	2.48 ± 3.82	.017
By the end of follow-up				
Follow-up time (months)	52 ± 18	44 ± 15	47 ± 19	.32
Overall remission rate, *n* (%)	37 (97)	12 (86)	13 (81)	.073
Remission rate by the end, *n* (%)	34 (89)	11 (79)	11 (69)	.17
Proteinuria (g/d)	1.70 ± 2.69	2.91 ± 3.62	3.13 ± 4.41	.028
Time-average proteinuria (g/d)	2.15 ± 1.69	3.84 ± 2.70	3.46 ± 3.19	.026
Reaching renal end point, *n* (%)	6 (16)	5 (36)	5 (31)	.23

Non-CsA-RI: no cyclosporine related renal injury; CsA-ARI: cyclosporine related acute renal injury; CsA-CRI: cyclosporine related chronic renal injury; CsA: cyclosporine; eGFR: estimated glomerular filtration rate; CKD: chronic kidney disease; IMN: idiopathic membranous nephropathy; RAS: renin angiotensin aldosterone system.

### Long term renal outcome and renal survival analysis

**Figure 3.**
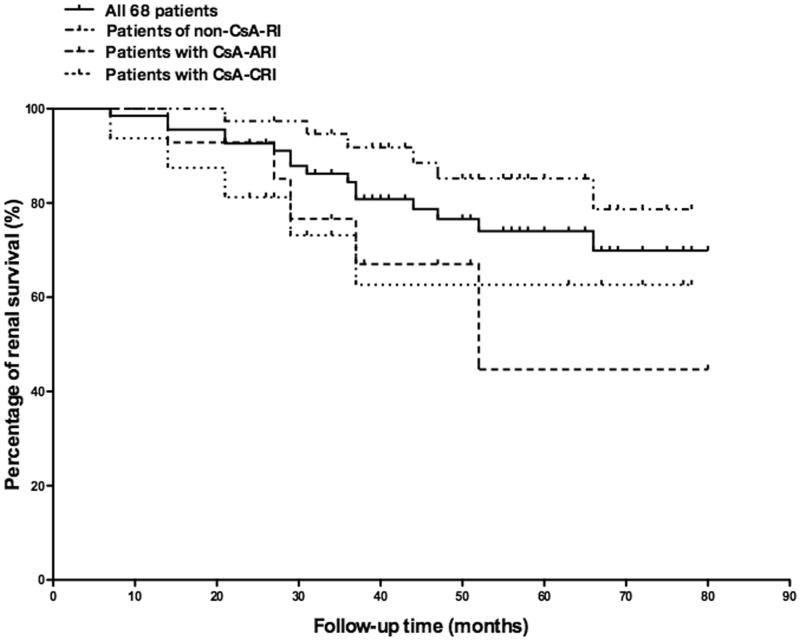
The cumulative renal survival percentage of all 68 patients, patients with CsA-ARI, patients with CsA-CRI and patients in Non-CsA-RI group.

By the end of follow-up, 16 patients (24%, 16/68) reached renal endpoint (Figure 3), among which 3 patients (4%, 3/68) who had refractory nephrotic syndrome developed end stage renal disease (ESRD) at 16, 48, and 74 months, respectively. By multivariate analysis, intimal sclerosis of small artery and TAP were identified as independent risk factors for reaching long-term renal endpoint, while RAS inhibition treatment was found to be an independent protective factor (Table 2). There was no association between the cumulative using time or dosage of cyclosporine and the development of renal endpoint. Patients with CsA-CRI had the lowest renal function at the end of follow-up, while those who had CsA-ARI presented the highest degree of renal function decline (Figure 2). Patients with CsA-ARI or CsA-CRI had higher proportion of cases reaching renal endpoint compared with those in the non-CsA-RI group, whereas the difference did not reach statistical significance (Table 1).

**Table 2.** Univariate and multivariate analysis for renal endpoint.

		95% CI		
	HR	Lower	Upper	*p*-Value
Univariate analysis				
Baseline data at CsA commencement				
Age (years)	1.035	0.994	1.078	.092
Gender (male vs. female)	0.740	0.268	2.039	.56
Serum creatinine (per µmol/L)	1.010	0.977	1.044	.56
eGFR (per ml/min/1.73 m^2^)	0.996	0.975	1.017	.69
CKD stage (per stage)	0.876	0.281	2.724	.82
Proteinuria (per g/d)	0.984	0.854	1.133	.82
Serum albumin (per g/L)	0.873	0.789	0.967	.009
Hypertension (yes vs. no)	1.499	0.536	4.191	.44
Diabetes mellitus (yes vs. no)	1.247	0.355	4.386	.73
Use of RAS inhibitors (yes vs. no)	0.196	0.071	0.544	.002
Renal pathology				
Tubule/interstitial acute index (per)	0.901	0.461	1.762	.76
Tubule/interstitial chronic index (per)	0.975	0.525	1.812	.94
Artery/arteriole lesions (yes vs. no)				
Intimal sclerosis of small artery	5.895	1.306	26.611	.021
Hyalinosis of arteriole	1.838	0.664	5.090	.24
Data during follow up				
Occurrence of CsA-ARI (yes vs. no)	2.171	0.744	6.330	.16
Occurrence of CsA-CRI (yes vs. no)	1.902	0.657	5.509	.24
Complete/partial remission (yes vs. no)	0.104	0.026	0.422	.002
Time average proteinuria (per g/d)	1.775	1.414	2.229	<.001
Duration of cyclosporine use (per month)	0.945	0.909	0.983	.005
Culmulative cyclosporine dose (per 100g)	0.422	0.148	1.205	.11
Multivariate analysis				
Age (per year)	1.031	0.975	1.090	.28
Gender (male vs. female)	2.864	0.638	12.856	.17
Baseline serum albumin (per g/L)	0.895	0.755	1.060	.20
Intimal sclerosis of small artery (yes vs. no)	5.178	1.011	26.532	.049
Use of RAS inhibitors (yes vs. no)	0.164	0.032	0.850	.031
Complete/partial remission (yes vs. no)	0.581	0.028	12.145	.73
Time average proteinuria (per g/d)	1.594	1.060	2.396	.025
Duration of cyclosporine use (per month)	0.993	0.949	1.039	.76

CsA: cyclosporine; eGFR: estimated glomerular filtration rate; CKD: chronic kidney disease; RAS: renin angiotensin aldosterone system; Non-CsA-RI: no cyclosporine related renal injury; CsA-ARI: cyclosporine related acute renal injury; CsA-CRI: cyclosporine related chronic renal injury.

As TAP was found to play important role in long-term renal outcome in our cohort, we then categorized patients into three groups based on the levels of TAP, i.e. <1.0 g/d, 1.0–3.5 g/d and >3.5 g/d. Patients with TAP of >3.5 g/d had the most prominent nephrotic status at baseline; were prescribed the highest dosage of cyclosporine; had the highest serum levels of cyclosporine but the lowest remission rate among the three groups. They presented the highest degree of eGFR decrease during the first three months and had the highest proportions of cases developed CsA-ARI or reached renal endpoint (Table 3).

**Table 3.** Comparison on baseline and overtime clinical-pathological data among patients with different time-average proteinuria.

		<1.0 g/d (*n* = 18)	1.0 ∼ 3.5 g/d (*n* = 30)	>3.5 g/d (*n* = 20)	*p* Value
Baseline data at CsA commencement					
Age (years)		52 ± 16	52 ± 12	56 ± 13	.54
Male, *n* (%)		8 (44)	18 (60)	12 (60)	.52
Mean blood pressure (mmHg)		90 ± 10	94 ± 9	96 ± 11	.35
Serum creatinine (µmol/L)		74.4 ± 13.9	7.6 ± 1.4	76.4 ± 22.2	.63
eGFR (ml/min/1.73 m^2^)		98.7 ± 22.3	106.5 ± 16.5	104.6 ± 36.6	.30
CKD stage 1:stage 2 (*n*)		10:8	26:4	11:9	.021
Serum albumin (g/L)		29.5 ± 5.6	28.2 ± 5.4	24.9 ± 6.5	.046
Proteinuria (g/d)		4.90 ± 2.63	6.37 ± 3.76	7.81 ± 3.90	.047
Hypertension, *n* (%)		7 (39)	9 (30)	7 (35)	.81
Diabetes mellitus, *n* (%)		4 (22)	1 (3)	5 (25)	.042
Renal pathology					
Stage of IMN (I:II + III) (*n*)		14:4	16:14	6:14	.013
Tubule/interstitial acute index		1.2 ± .8	1.2 ± .8	1.2 ± .6	1.00
Tubule/interstitial chronic index		1.3 ± .8	1.2 ± .8	1.4 ± .7	.72
Artery/arteriole lesions, %(*n*/*n*)					
Intimal fibrosis of small artery		35 (6/17)	57(17/30)	58 (11/19)	.30
Hyalinosis of arteriole		39 (7/18)	38 (11/29)	25 (5/20)	.58
Treatment					
RAS inhibition, *n* (%)		16(89)	27 (90)	14 (70)	.17
Initial prednisone dose (mg/kg/d)		0.3 ± 0.2	0.3 ± 0.2	0.3 ± 0.2	.90
Initial CsA dose (mg/kg/d)		1.4 ± 0.4	1.6 ± 0.4	1.7 ± 0.5	.15
Maximum CsA dose in first 3 m (mg/kg/d)		1.8 ± 0.5	2.1 ± 0.5	2.2 ± 0.6	.095
Peak serum CsA level in first 3 m (ng/ml)		87.04 ± 45.20	11.36 ± 43.63	112.48 ± 51.03	.075
Data at 3rd month					
Serum creatinine (µmol/L)		77.2 ± 21.4	78.0 ± 17.6	9.6 ± 18.4	.046
eGFR (ml/min/1.73 m^2^)		99.4 ± 34.7	96.7 ± 22.0	8.9 ± 21.7	.056
ΔeGFR (ml/min/1.73 m^2^)		1.4 ± 29.6	13.6 ± 27.4	23.5 ± 24.8	.292
Serum albumin (g/L)		36.5 ± 3.8	32.3 ± 5.5	29.34 ± 6.8	.002
Proteinuria (g/d)		1.81 ± 1.50	3.98 ± 2.58	6.18 ± 3.12	<.001
Remission rate, *n* (%)		14 (78)	12 (40)	3 (15)	<.001
Data at the end of follow up					
Follow-up time (months)	57 ± 15		53 ± 15	38 ± 19	.001
Duration of cyclosporine treatment (months)	33 ± 17		36 ± 15	22 ± 18	.018
Serum creatinine (µmol/L)	89.7 ± 15.9		102.1 ± 32.2	168.5 ± 132.4	.013
eGFR (ml/min/1.73 m^2^)	8.29 ± 17.80		72.08 ± 19.76	54.81 ± 26.51	.002
ΔeGFR (ml/min/1.73 m^2^)	18.4 ± 15.3		34.4 ± 19.4	51.5 ± 32.2	.001
CKD stage 1:stage 2:stage 3:stage 4 (*n*)	7:7:4:0:0		7:15:7:1:0	2:8:6:1:3	.39
Serum albumin (g/L)	41.59 ± 4.00		37.49 ± 8.79	32.73 ± 7.78	.001
Proteinuria (g/d)	0.30 ± .48		1.50 ± 2.06	4.30 ± 4.22	<.001
Remission rate, *n* (%)	18 (100)		25 (83)	13 (65)	.017
Reaching renal endpoint, *n* (%)	0 (0)		7 (23)	9 (45)	.002
Follow up overtime data					
Patients with CsA-ARI, *n* (%)		2 (11)	3 (10)	9 (45)	.009
Patients with CsA-CRI, *n* (%)		3 (17)	7 (23)	6 (30)	.67
Overall remission rate, *n* (%)		18 (100)	30(100)	14 (70)	.001
Time-average proteinuria (g/d)		0.60 ± .24	1.95 ± .66	5.38 ± 1.93	<.001

CsA: cyclosporine; eGFR: estimated glomerular filtration rate; CKD: chronic kidney disease; IMN: idiopathic membranous nephropathy; RAS: renin angiotensin aldosterone system; CsA-ARI: cyclosporine related acute renal injury; CsA-CRI: cyclosporine related chronic renal injury.

### Extra-renal adverse events

Extra-renal adverse events included infections (9%), gingival hyperplasia (4%), neurological symptoms (4%), hypertension (3%), gastrointestinal symptoms (3%), elevated liver enzymes (2%), cardiovascular events (3%), anemia (2%), skeleton muscle symptoms (2%), skin symptoms (2%) and diabetes mellitus (3%). Cyclosporine was discontinued in two cases for the reason of anemia and anxiety, respectively.

## Discussion

Cyclosporine has been used to treat IMN for decades with high remission rate, whereas two issues remain unresolved including cyclosporine associated renal injury, which is in a dose-dependent way as reported [21–23], and high relapse after discontinuing the medicine [6,10,24] which leads to a prolonged treatment strategy. The present study aimed to evaluate the effectiveness and renal safety of a low-dose long-standing course of cyclosporine in patients with IMN.

The maximum dosage of cyclosporine in the current prospective nephrotic IMN cohort was 2.0 ± 0.5 mg/kg/d with a whole trough cyclosporine blood level of 105.2 ± 46.8 ng/ml, which was much lower than what was recommended by KDIGO guideline (3 to 5 mg/kg/d to reach a blood level of 125–175 ng/ml) [16]. The overall remission rate was 91% with PR reached at 4.5 (1–12) months and CR at 12.5 (4–49) months, which was comparable to the previous reports in which a 46.4%∼94.1% of remission rate was achieved at a cyclosporine dose of 3.46 ± 0.63 ∼ 3.7 ± 2.0 mg/kg/d by 12 months [6,9,15,24–26], and a 57.1–100% of remission rate at a cyclosporine dose of 2 mg/kg/d (trough cyclosporine blood level of 80–120 ng/ml) [11,12,14]. Although the recurrence rate in our cohort (42%) was comparably high as reported (43–47%) [6,10,24], most of the recurrent cases (70%) responded again to cyclosporine treatment. By the end of follow-up, the long-term cyclosporine treatment (median 27 months) maintained a remission rate of 82%, which is relatively high considering such long follow-up time (average 50 months). Therefore, the lower dose and long-standing cyclosporine is effective in treating patients with IMN, with similar remission rate as the KDIGO recommended regimen and meanwhile has no increase in relapse rate.

However, with this long-standing follow-up time, we found that nearly 1/4 patients reached renal endpoint, which raised our concerns about the renal safety issue in this IMN cohort. Since it has been well acknowledged that persistent high proteinuria increases the risk of chronic renal failure [4,27,28], we checked the effects of proteinuria on the long-term renal outcome. In the current cohort, three patients with refractory nephrotic syndrome developed ESRD within 1.5–6 years and TAP was an independent risk factor for worse long-term renal outcome. These data emphasize the critical role of proteinuria in disease progression and reinforce the importance of implementing effective treatment targeting proteinuria in IMN. However, will the patients with uncontrolled proteinuria benefit from higher dose of cyclosporine? Apart from proteinuria, did cyclosporine also contribute to the impairment of renal function? Moreover, is there any simple way to recognize patients that might have poor benefit/risk ratio with cyclosporine at early stage? These are the questions need to be answered.

In our cohort, patients with TAP of >3.5 g/d were more nephrotic at commencement, tended to be treated with higher initial and maximum cyclosporine doses, and achieved relatively higher cyclosporine blood levels, whereas the remission rate was much lower than patients with less TAP, which implies that they were less responsive to cyclosporine and might need a higher dose. However, these patients had developed prominent decrease in renal function with 45% developed CsA-ARI and 30% had CsA-CRI. This rapid change in renal function after cyclosporine treatment would rather be attributed to cyclosporine than blame the progression of glomerular diseases, and obviously does not favor increasing cyclosporine dosage.

In this study, cyclosporine dose or blood level were not associated with CsA-ARI or CsA-CRI, which indicate that the renal toxicity of low-dose cyclosporine in this Chinese cohort of IMN patients does not show the feature of dose dependent, but rather be relevant to individual susceptibility. Meanwhile, the cumulative using time or dosage of cyclosporine, or the occurrence of CsA-ARI or CsA-CRI, was not associated with renal endpoint, which appeared as if cyclosporine and cyclosporine-associated renal injury did not contribute to the long-term renal impairment. This may be because cyclosporine induced renal injury was mostly reversible when we adjusted the dosage or discontinued the treatment of cyclosporine in time, so that the transient renal injury induced by cyclosporine would not affect the long-term renal outcome in our cohort.

Our study suggests that in patients of older age, with lower baseline eGFR levels, or having intimal sclerosis of small artery, careful monitor of renal function should be performed when low dose of cyclosporine was prescribed. Besides, patients who developed CsA-ARI had the highest TAP as well as the highest proportion of patients (36%) reaching renal endpoint, and therefore, had the lowest benefit/risk ratio among the three groups. Based on these data, it is reasonable to discontinue cyclosporine and switch to a non-calcineurin-inhibitor based regimen in patients with CsA-ARI particularly in those who have not got remission by the 3rd month, rather than continue to observe till 6 months. Patients who developed CsA-CRI also had higher TAP and a higher proportion reaching renal endpoint (31%) compared with non-CsA-RI group. In those who have developed CsA-CRI but still not achieved remission or had recurrence, a switch to non-calcineurin-inhibitor based regimen should also be considered. In addition, RAS inhibition was found to be independently protective against poor long-term renal outcome and not related to either CsA-ARI or CsA-CRI, which strengthens the usage of RAS inhibition in the treatment of nephrotic IMN.

Our study has limitations: (1) this was a small sample size and uncontrolled study; (2) no biomarkers were tested to evaluate renal tubule injuries, and might lose useful information for predicting cyclosporine-related renal injury; (3) no repeat renal biopsy were taken to confirm the suspected chronic cyclosporine related renal injury. Despite all the limitations, our study is one of the few studies reporting the use of low-dose and long-term cyclosporine for patients with nephrotic IMN. Moreover, this is the first study specifically describing the renal function change over time during cyclosporine treatment, the affecting factors and its related clinical significance, which provides valuable information for clinicians.

## Conclusions

Low-dose cyclosporine is effective in treating IMN. However, cyclosporine-associated renal injury remains an unneglectable concern at this low-implemented dosage. Acute increase in SCr and no response in proteinuria during the first three months of cyclosporine treatment is a strong indicator for low renal benefit/risk ratio, and these patients should be switched to non-calcineurin-inhibitor based regimen. Patients of older age, with lower baseline eGFR, or having intimal sclerosis of small artery, are more likely to develop progressive renal dysfunction.
